# Long-term continuous treatment of non-sterile real hospital wastewater by *Trametes versicolor*

**DOI:** 10.1186/s13036-019-0179-y

**Published:** 2019-05-29

**Authors:** Josep Anton Mir-Tutusaus, Eloi Parladé, Marta Villagrasa, Damià Barceló, Sara Rodríguez-Mozaz, Maira Martínez-Alonso, Núria Gaju, Montserrat Sarrà, Glòria Caminal

**Affiliations:** 1 Departament d’Enginyeria Química Biològica i Ambiental, Escola d’Enginyeria, Universitat Autònoma de Barcelona, Bellaterra, 08193 Barcelona, Spain; 2 Departament de Genètica i Microbiologia, Universitat Autònoma de Barcelona, Bellaterra, 08193 Barcelona, Spain; 3 Catalan Institute for Water Research (ICRA), Scientific and Technological Park of the University of Girona, H2O Building, Emili Grahit 101, 17003 Girona, Spain; 4 Department of Environmental Chemistry, Institute of Environmental Assessment and Water Research (IDAEA), Spanish Council for Scientific Research (CSIC), Jordi Girona 18-26, 08034 Barcelona, Spain; 5 Institut de Química Avançada de Catalunya (IQAC) CSIC, Jordi Girona 18-26, 08034 Barcelona, Spain

**Keywords:** Fungal bioreactor, Pharmaceutical active compounds, Microbial community, Hospital wastewater, Pretreatment

## Abstract

**Background:**

Hospital wastewater is commonly polluted with high loads of pharmaceutically active compounds, which pass through wastewater treatment plants (WWTPs) and end up in water bodies, posing ecological and health risks. White-rot fungal treatments can cope with the elimination of a wide variety of micropollutants while remaining ecologically and economically attractive. Unfortunately, bacterial contamination has impeded so far a successful implementation of fungal treatment for real applications.

**Results:**

This work embodied a 91-day long-term robust continuous fungal operation treating real non-sterile hospital wastewater in an air pulsed fluidized bed bioreactor retaining the biomass. The hydraulic retention time was 3 days and the ageing of the biomass was avoided through partial periodic biomass renovation resulting in a cellular retention time of 21 days. Evolution of microbial community and *Trametes* abundance were evaluated.

**Conclusions:**

The operation was able to maintain an average pharmaceutical load removal of over 70% while keeping the white-rot fungus active and predominant through the operation.

**Electronic supplementary material:**

The online version of this article (10.1186/s13036-019-0179-y) contains supplementary material, which is available to authorized users.

## Background

Pharmaceutically active compounds (PhACs) occurrence in water bodies remains an issue of environmental concern despite persistent efforts of the scientific and global community to cope with the problem. PhACs can be found at relevant concentrations in the environment and can pose a wide range of risks to the ecosystem and human health [1–3]. Wastewater treatment plants (WWTPs) are the primary source of PhACs into the environment, since they are not designed nor operated to remove these micropollutants [4]. Hospital wastewater (HWW) contains higher concentrations of PhACs, which make hospitals a good target for on-site treatment [5]. Otherwise, HWW is usually discharged untreated to the sewer network, thus contributing to the PhACs loads into WWTP influent [3].

Removing such micropollutants from water streams is not trivial, although many advances have been achieved in that direction. While many studies have dealt with the degradation of single pollutants, real streams have mixtures of several contaminants, thus a robust process should be capable of coping with the removal of most micropollutants. Amongst the biological oxidation processes, white-rot fungi (WRF) have proved to be especially well-suited for removing and degrading a wide range of pharmaceuticals [6, 7]. A versatile enzymatic system comprising both intracellular (e.g., cytochrome P450 system) and extracellular enzymes (e.g., laccase-mediators system) allows these fungi to transform most of the PhACs, usually very recalcitrant, to more biodegradable compounds or even achieve complete mineralization [8, 9]. Therefore, a fungal system has been regarded as a feasible approach for pretreating hospital effluents prior to discharge to the WWTP, as some pharmaceuticals would be removed and other transformed into more biodegradable compounds, suitable for the posterior conventional activated sludge treatment (CAS) [10]. This approach suggests a single fungal process that can remove most pharmaceutical load, rather than using a specific treatment for every compound. However, full scale applications of this technology do not exist at the moment. Developments in this direction depend on overcoming several shortcomings, namely: (1) maintaining a stable activity of the fungal pellets over prolonged periods of time and (2) preserving good performance in non-sterile conditions, as sterility would be unviable from the economic and ecological perspective. On one hand, removal efficiency can be higher in non-sterile matrices than in sterile conditions due to the consortium established [11]. Additionally, in non-sterile matrices bacteria could degrade the most biodegradable transformation products of the xenobiotic parent compounds transformed by the WRF [12]. On the other hand, non-sterility reduces the duration of bioreactor operation due to native microorganisms exerting competitive pressure in WRF survival. This aspect has been partly resolved by introducing a pretreatment step that reduces the initial concentration of microorganisms in the influent [13]. A partial biomass regeneration strategy could also help to increase the duration of the bioreactor operation [14]. In any case, the interactions between the inoculated fungus and the microbial community existing in the wastewater and developed during the treatment seem to be case-dependent and are highly complex. This fact calls for a thorough study of the microbial communities developed during a more stable, long-term operation. Furthermore, identification of native HWW PhACs degraders could lead to a better understanding of the process and improvement in the reactor operation.

Some studies have optimized values for pH, temperature, growth conditions, aeration, pellet size, biomass renovation and nutrients addition [14–16]. Those previous studies enabled this long-term operation of a fungal fluidized bed bioreactor treating real non-sterile wastewater for the first time. The objectives of the study were to prove the concept of a long-term fungal treatment of real HWW, evaluating the bacterial and fungal communities arisen during the treatment and monitoring the abundance of the inoculated fungi to assess the removal efficiency for PhACs.

## Methods

### Reagents, fungus and hospital wastewater

Thiamine hydrochloride was acquired from Merck (Barcelona, Spain) and glucose, ammonium chloride and other chemicals were purchased from Sigma-Aldrich (Barcelona, Spain). Primers were acquired from Invitrogen (Barcelona, Spain) and quantitative PCR supermix from Bio-Rad (Barcelona, Spain). All other chemicals used were of analytical grade. Coagulant Hyfloc AC50 and flocculant Himoloc DR3000 were kindly provided by Derypol, S.A. (Barcelona, Spain).

*T. versicolor* (ATCC#42530) was maintained on 2% malt agar slants at 25 °C until use. Subcultures were routinely made. A mycelial suspension of *T. versicolor* was traditionally obtained as previously described elsewhere [14]. After the startup, the mycelial suspension was obtained with the novel method by homogenization of pelleted biomass described elsewhere [16].

The HWW was collected from the sewer manifold of Sant Joan de Déu Hospital (Barcelona, Catalonia) in the NE of Spain. The physic-chemical characteristics of the wastewater are summarized in Additional file 1: Table S1.

### Medium and pellet growth

Fungal pellets of 2 ± 1 mm of diameter were obtained as previously described by Borràs et al. (2008) [15] by inoculating 20 mL·L^− 1^ of the mycelial suspension in 2 L of a defined medium in a sterile glass air-pulsed fluidized bioreactor. The medium contained, per liter: 7 g glucose, 100 mL macronutrients, 10 mL micronutrients, 2.1 g NH_4_Cl and 10 mg thiamine [15]. The pH was controlled at 4.5 by adding HCl 1 M or NaOH 1 M, the O_2_ was measured and the temperature was maintained at 25 °C; pH and temperature profiles can be found in Additional file 1: Figure S1. Air pulses (1 s air pulse every 4 s) provided 0.8 L·min^− 1^ pulsed aeration and fluidization to the reactor.

### HWW treatment

Hospital wastewater was pretreated with a coagulation-flocculation process (38 mg·L^− 1^ of coagulant and 3.3 mg·L^− 1^ of flocculant) as described previously [13]. In order to have a constant influent during all the experiment, the flocculated wastewater was distributed in bottles and stored at − 20 °C. This methodology will help the interpretation of the the results.

After the pellet growth, the pellets were retained in the 2 L reactor, the medium was withdrawn and the reactor filled with pretreated HWW. The fluidized bed reactor operated continuously with a hydraulic residence time (HRT) of 3 days. A partial biomass renovation strategy described by Blánquez et al. (2006) produced a cellular retention time (CRT) of 21 d [14]. Glucose and NH_4_Cl were added to the reactor at consumption rate, with a carbon-to-nitrogen ratio of 7.5 (mol/mol). Samples were withdrawn periodically for analysis.

### Analysis of pharmaceuticals

Samples from the effluent were removed weekly and analyzed per triplicate following the analytical procedure described elsewhere [5]. A list of 74 PhACs were analysed. This can be seen in Additional file 1: Table S2. Briefly, 10 mL of sample for raw HWW and 25 mL for treated HWW were pre-concentrated by Solid Phase Extraction as previously described [17]. Elution was done with 6 mL of pure methanol. The extracts were evaporated under nitrogen stream and reconstituted with 1 mL of methanol-water (10:90 v/v). 10 μL of standard of internal standard mix at 1 ng·μL^− 1^ were added for calibration. Separation was carried out using an Ultra-Performance liquid chromatography system (Waters Corp. Milford, MA, USA), equipped with an Acquity HSS T3 column (50 mm × 2.1 mm i.d. 1.7 μm) for the compounds analyzed under positive electrospray ionization and an Acquity BEH C18 column (50 mm × 2.1 mm i.d., 1.7 μm) for the ones analyzed under negative electrospray ionization, both from Waters Corporation. The UPLC was coupled to a 5500 QqLit, triple quadrupole–linear ion trap mass spectrometer (5500 QTRAP, Applied Biosystems, Foster City, CA, USA) with a Turbo V ion spray source. Two MRM transitions per compound were recorded by using the Scheduled MRM™ algorithm and the data were acquired and processed using Analyst 2.1 software.

### Microbial community analysis

Pelleted biomass samples from the reactor were centrifuged at 14.000 rpm and pellets were freeze-dried and stored at − 80 °C. Liquid samples from the reactor were filtered through 0.22 μm GV Durapore® membrane filters (Merck Millipore, USA) and filters were stored at − 80 °C. Total DNA extraction, PCR amplification and denaturing gradient gel electrophoresis (DGGE) were conducted as previously described [17].

Bands from the gel were excised, reamplified and then sequenced by Macrogen (Madrid, Spain). Sequences were trimmed and checked for chimeras using Mothur [18]. Each 16S rRNA sequence was assigned to its closest neighbor according to the Basic Local Alignment Search Tool (BLAST) results [19]. Representative sequences of each band were deposited in GeneBank under the accession numbers MF682288 to MF682321 and MF683211 to MF683229 for bacteria and fungi, respectively.

The qPCR assay was performed using a specific primer set targeting *T. versicolor* [20] and the following amplification protocol: 95 °C for 10 min and 40 cycles of 95 °C for 15 s and 60 °C for 1 min. Reactions contained 1× ssoAdvanced Universal SYBR Green Supermix (Bio-Rad), 0.3 μM of each forward and reverse primers and 12 ng of sample DNA in a final volume of 20 μL. All reactions were run in triplicate in a CFX95 Real-Time System (Bio-Rad) with a resulting amplification efficiency of 96.5% and R^2^ value of 0.999. A calibration curve was prepared using a purified amplicon (302 bp) from the Internal Transcribed Spacer (ITS) region of *T. versicolor* ATCC 42530.

### Other analyses

Turbidity was determined as the absorbance at 650 nm by a UNICAM 8625 UV/VIS spectrometer and the conductivity by a CRISON MicroCM 2100 conductometer. Chloride, sulfate, nitrate and phosphate anions were quantified by a Dionex ICS-2000 ionic chromatograph. The total suspended solids (TSS) were determined according to the Standard Methods for the Examination of Water and Wastewater [21]. Ammonia concentration and chemical oxygen demand (COD) were analyzed by using commercial kits LCK114 or LCK314, respectively (Hach Lange, Germany). Glucose concentration was measured with a YSI 2700 SELECT enzymatic analyzer (Yellow Spring Instruments). Laccase activity was measured per duplicate using a modified version of the method for the determination of manganese peroxidase where 2,6-dimetoxyphenol (DMP) is oxidized by laccase in the absence of a cofactor [22]. Activity units per liter (U·L^− 1^) are defined as the micromoles per liter of DMP oxidized per minute. The molar extinction coefficient of DMP was 24.8 mM^− 1^·cm^− 1^ [23].

### Statistical analysis

A robust Regression on Order Statistics (ROS) approach was used for dealing with left-censored data, namely: below limit of detection (BLD) and below limit of quantification (BLQ) values [24]. When ROS was not possible, values BLD and BLQ were considered to have a concentration $\mathrm{BLD}/\sqrt{2}$ and $\mathrm{BLQ}/\sqrt{2}$, respectively. Data analysis was performed with R: A language and environment for statistical computing. ROS was performed using the R package NADA [25].

## Results and discussion

Physicochemical characteristics of the wastewater used are summarized in Additional file 1: Table S1. All values fell within the range of similar wastewaters, either from the same hospital or from other hospitals of the same region [13, 17]. The approximately 75% decrease of both TSS and COD after flocculation was also reported previously. The wastewater was taken from a hospital with an important psychiatric ward; therefore, high levels of psychiatric drugs were detected. Out of 74 compounds analyzed, 45 were detected and from those, 11 belonged to the psychiatric drugs group. The PhACs concentration before and after the coagulation-flocculation pretreatment of the HWW are summarized in Additional file 1: Table S2. A total concentration of 7 μg·L^− 1^ psychiatric drugs was measured, being carbamazepine and its transformation products (2-OH-CBZ, acridone, epoxy-CBZ) and venlafaxine and their transformation products (TPs) (N-desmethyl-venlafaxine and O-desmethyl-venlafaxine) the most abundant ones. The presented psychiatric pharmaceuticals concentration, around 15.4% of the initial load was substantially lower than in other psychiatric hospitals [26], but higher than other founded in general hospitals [27]. As it is common in HWW, the analgesics and anti-inflammatories family contributed the most to the overall pharmaceutical load (75% in this study) [12, 28], with ibuprofen, salicylic acid (a transformation product of aspirin) and acetaminophen (also known as paracetamol), in that order, being the most important of the group.

### Reactor performance

**Fig. 1:**
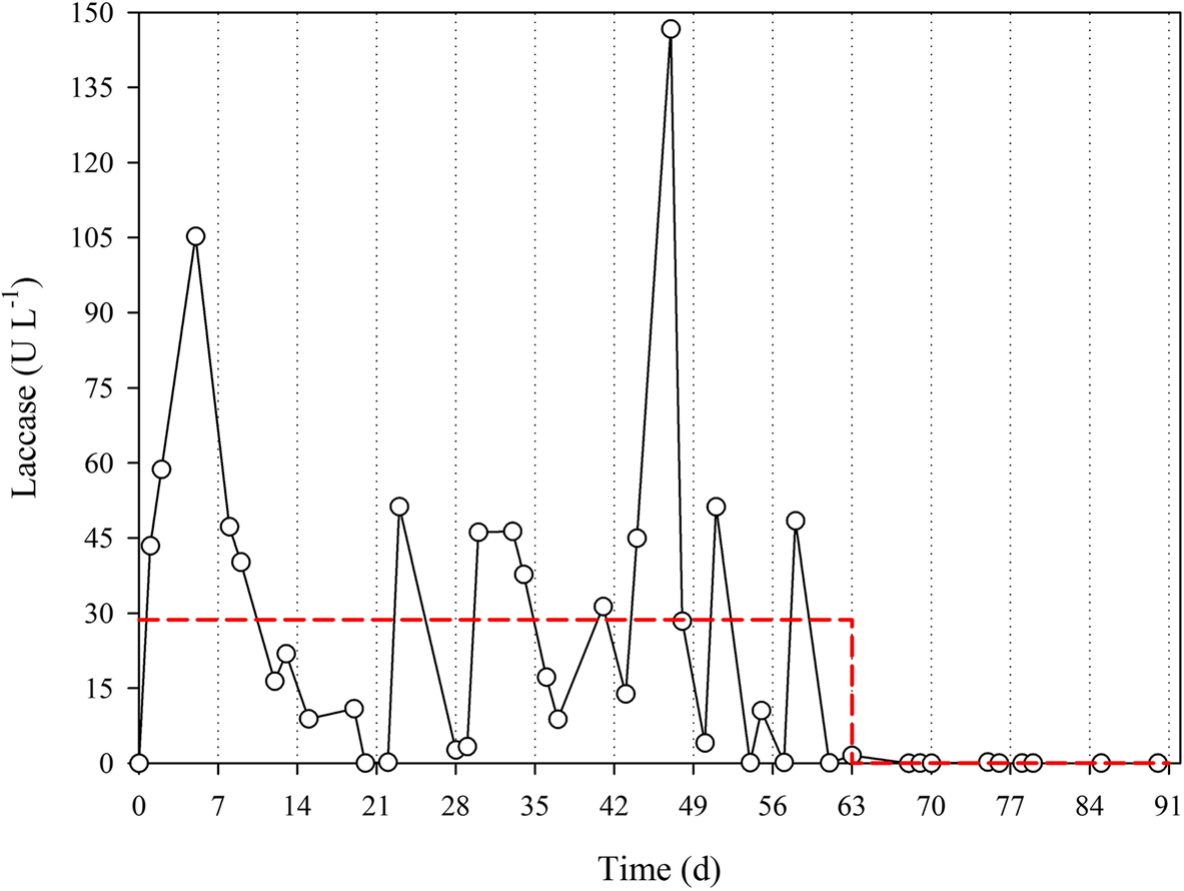
Evolution of laccase activity during the continuous treatment. Vertical dotted lines represent the weekly partial biomass regeneration. The red dashed line represents the average value in a pseudo steady state

Reactor performance was evaluated through the monitoring of laccase activity and glucose concentration along the treatment as a measure of fungal activity; since real-time determination of micropollutant concentration is not feasible. Laccase activity profile during the operation is presented in Fig. 1. Two periods could be observed. The first, before 63th day, where laccase is detected around 30 U·L^− 1^ in a pseudo steady state. The second period, after 63th day, extracellular laccase activity levels remainedinsignificant. Although laccase activity was detected and glucose concentration was very low, the reactor gained a pinkish color between days 33 and 37, when reactor biomass was replaced by fresh pellets. As the initial week of process, the highest peaks of laccase were detected. Laccase activity could be linked to *Trametes* activity, but the low laccase activity detected at the end of the treatment could not be linked to the inactivation of the fungus, as demonstrated previously [13]. In fact, *T. versicolor* was maintained throughout the operation even when laccase could not be detected (discussed in section Evolution of bacterial and fungal populations in the bioreactor) thus confirming the non-correlation between absence of laccase activity and fungal inactivation. As glucose was added at consumption rate, glucose concentration remained insignificant during the treatment.

### Pharmaceutical load removal

**Fig. 2:**
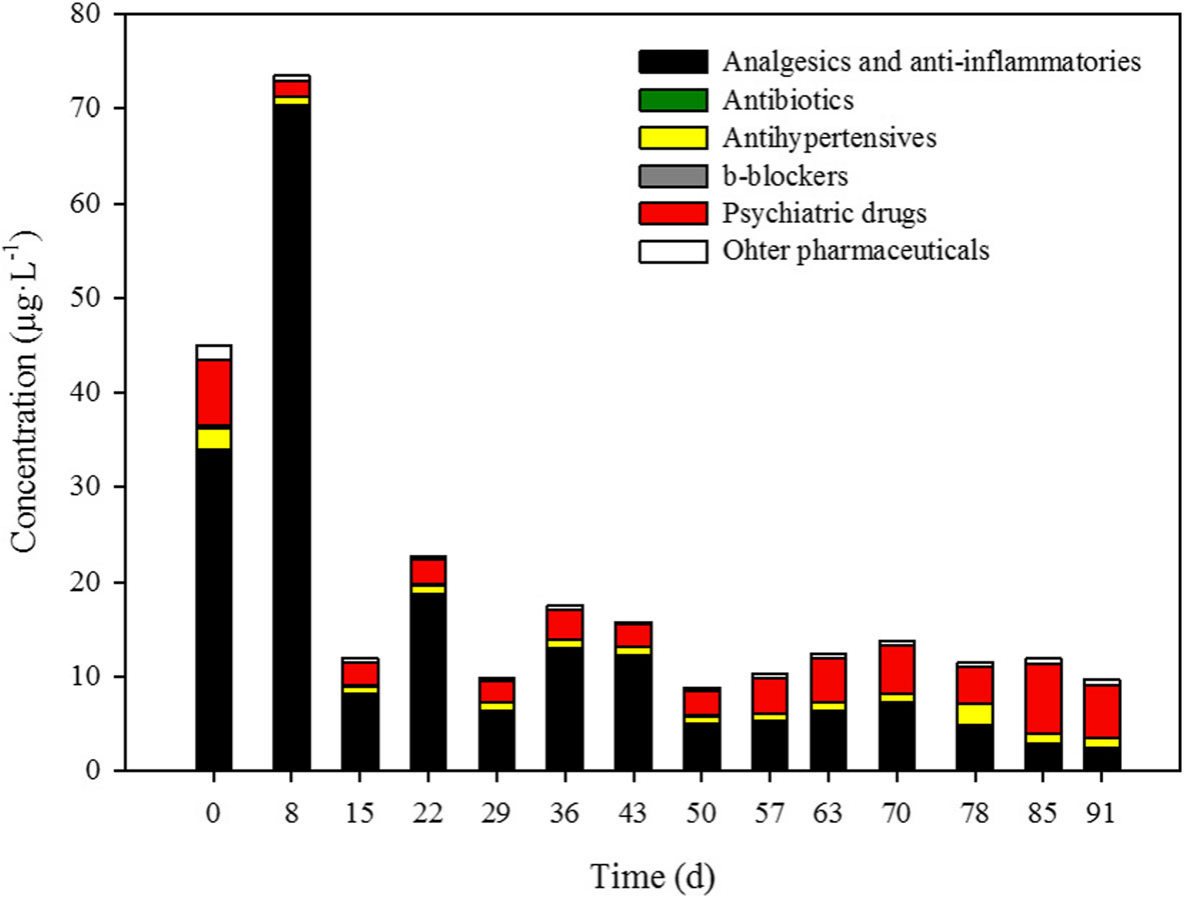
Concentration profile of pharmaceutically active compounds during the treatment

Time-course profile of concentrations of PhACs is presented in Fig. 2, organized by families. The initial concentration of PhACs was around 45 μg·L^− 1^, with the main contribution of analgesics and anti-inflammatories (75.0%), followed by psychiatric drugs (15.4%), antihypertensives and other pharmaceuticals. The increase of analgesics and anti-inflammatories concentration at Day 8 is mainly attributed to the high levels of ketoprofen and naproxen. Although *T. versicolor* has proved to degrade ketoprofen and naproxen both in sterile, defined media and in non-sterile HWW [8, 13], it was also hypothesized that it was able to deconjugate conjugated forms of ketoprofen and naproxen [17, 28, 29]. Briefly, conjugation is a chemical transformation performed in the liver in humans used to detoxify xenobiotics. It enables xenobiotics to be more water-soluble and excreted through urine. These conjugated compounds are present in the hospital wastewater as a result of human metabolism and can be cleaved by *T. versicolor.* This could explain the increase in the concentration of PhACs at Day 8. This fact established that the PhAC concentration in the influent was equal or higher than at Day 8; namely, that initial concentrations may be being underestimated. In any case the performance is still transitory because the steady state has not been reached.

From the second week of operation onwards the operation was considered a pseudo-steady state, given that 5 times the HRT had passed: as can be observed in Fig. 2, the global pharmaceutical concentration remained constant since Day 15, representing an overall 70% removal. The treatment is carried out under non-sterile conditions, so from the point of view of other microorganisms, bacteria and fungi different from *T. versicolor*, it should not be considered the steady state as a constant evolution of these populations was revealed through a DGGE + sequencing approach (section Evolution of bacterial and fungal populations in the bioreactor). During all treatment period, the treatment successfully removed pharmaceutical active compounds from the real non-sterile wastewater in a long-term operation. Total PhAC concentration after treatment was maintained around 15 μg·L^− 1^, although groupsdifferent pharmaceutical classes present a variable behaviour. For example: concentration of analgesics and anti-inflammatories decreased with time, probably due to an adaptation of native wastewater analgesics and anti-inflammatories degraders, as was concluded in a previous study [17]; in contrast, removal capacity of the psychiatric drugs group declined. This latter event is further discussed in section Carbamazepine and venlafaxine removal and transformation products.

Based on the profile of laccase activity and PhAC removal, it could be concluded that laccase was not essential to achieve PhAC degradation. Actually, degradation pathways in WRF are quite diverse: although laccase seems to play an important role in PhAC degradation [30], cytochrome P450 mechanism has been pointed out as the main transformation mechanism for several micropollutants [7, 8]. Therefore, in similar systems, drop in laccase activity does not necessarily indicate a decrease in PhACs removal efficiency. However, high concentrations of psychiatric drugs were found by the end of the treatment, when laccase activity was not detected. This aspect is further discussed in section Carbamazepine and venlafaxine removal and transformation products.

The mycelial suspension was prepared by homogenization of pelleted biomass, as proposed previously [16], and was used throughout the long-term operation. This novel method could substantially reduce the ecological and economic burden of mycelial preparation an industrial scale, and together with the use of low-cost medium, the technology could become more attractive economically.

**Fig. 3:**
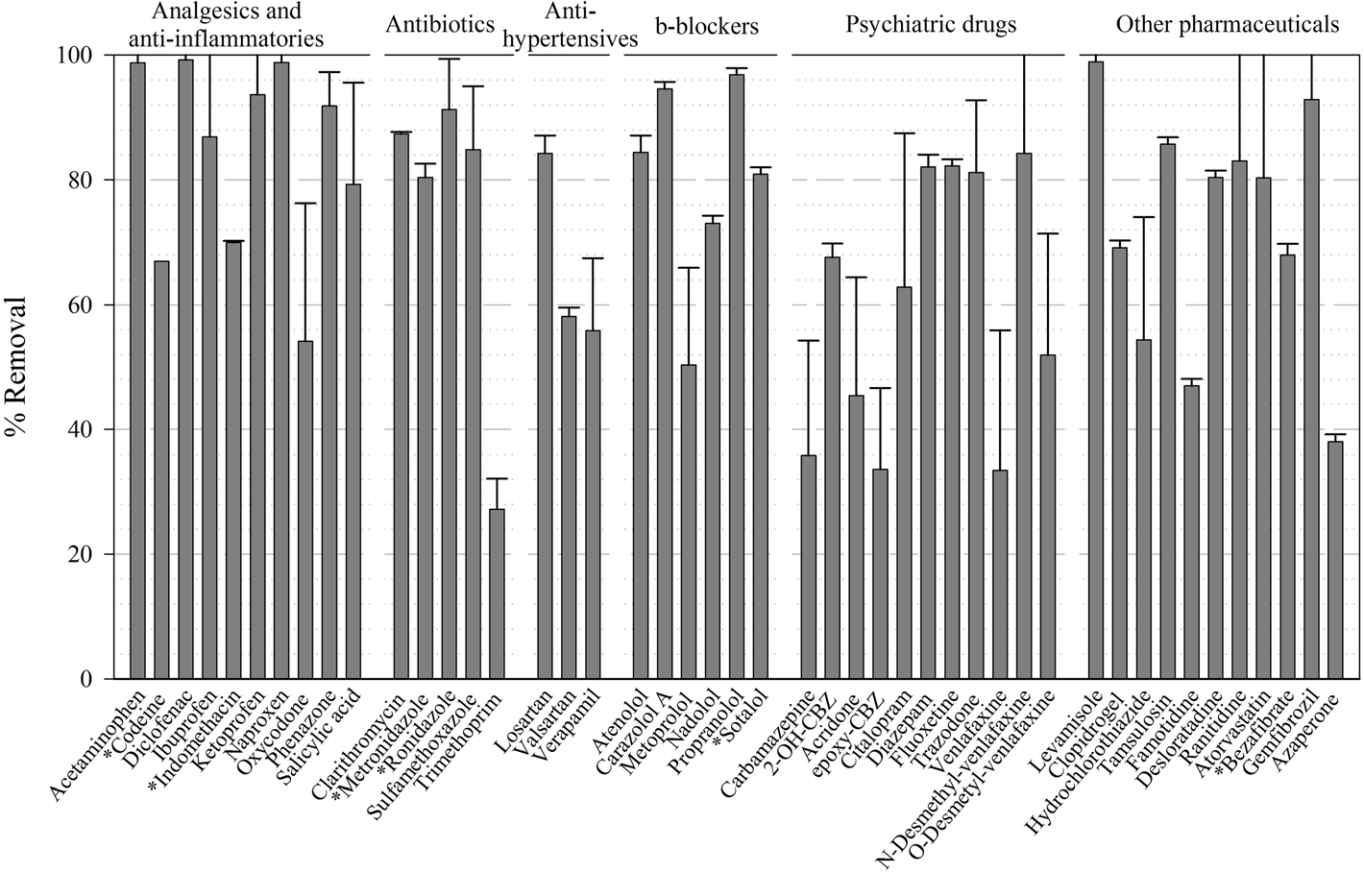
Removal values of pharmaceutically active compounds. Error bars represent the standard deviation of 12 samples during the steady state. Asterisks mark compounds whose initial concentration contained > 80% of left-censored data

The average removal of compounds during the steady state period (from Day 15 onwards) is presented in Fig. 3. Most analgesics and anti-inflammatories were removed over 80%, or more if we take into account deconjugation phenomena because the initial concentration was underestimated due to this phenomenon as it was explained before. Oxycodone was similarly removed (c.a. 60%) in a previous batch fungal treatment [28], while removal yield in activated sludge varies between 0 and 50% [31]. Diclofenac, ibuprofen and naproxen, all classified by the Global Water Research Coalition (GWRC) as high priority pharmaceuticals [32], were completely removed by the treatment. Diclofenac, in particular, a widely used anti-inflammatory drug, is usually quite recalcitrant in WWTPs [3].

Antibiotics were also removed over 80% with the exception of trimethoprim. Also low removal values for trimethoprim were achieved in previous studies with fungi (36–50% [17, 28]) as well as with activated sludge and even with anaerobic/anoxic/aerobic and UV processes in WWTPs [31, 33]. The other detected antibiotics are usually poorly or not removed in CAS and membrane bioreactor (MBR) systems [6], so the fungal treatment emerges as an interesting alternative. In fact, this long-term treatment slightly improved clarithromycin and sulfamethoxazole removal, but greatly improved ronidazole removal in comparison with shorter continuous and batch fungal operations (from 0 to 30 to > 90%) [17, 28].

β-blockers were removed between 50 and > 95%. These families included atenolol > metoprolol = propranolol > sotalol, listed in order of priority in GWRC report [32]. Considering that atenolol is the most abundant, the global β-blockers removal is over 80%. Eleven psychiatric drugs were detected in the hospital effluent: carbamazepine and its 3 TPs and venlafaxine and its 2 TPs are discussed in detail in section Carbamazepine and venlafaxine removal and transformation products. Citalopram was removed around 60% whereas diazepam removal (82%) was much higher than previous removal efficiencies (0–50%), achieved in shorter-term operation and batch studies [17, 28]. The removals of fluoxetine and trazodone were also very high, around 82 and 81%, respectively.

In regards to the miscellaneous group, anthelmintic levamisole, antiplatelet agent clopidogrel, drug against prostatic hyperplasia tamsulosin, H1 and H2 receptor antagonists desloratadine and ranitidine, and lipid regulators atorvastatin, bezafibrate and gemfibrozil were removed around 70% or above, whilst diuretic hydrochlorothiazide, H1 and H2 receptor antagonist famotidine and veterinary tranquilizer azaperone were removed around 50% or below. In general, removal values for all groups were higher than in previous short-term studies [17, 28] probably due to the prior optimization of carbon-to-nitrogen ratio in the nutrients addition, aeration, biomass renovation and pellet size [16].

### Carbamazepine and venlafaxine removal and transformation products

**Fig. 4:**
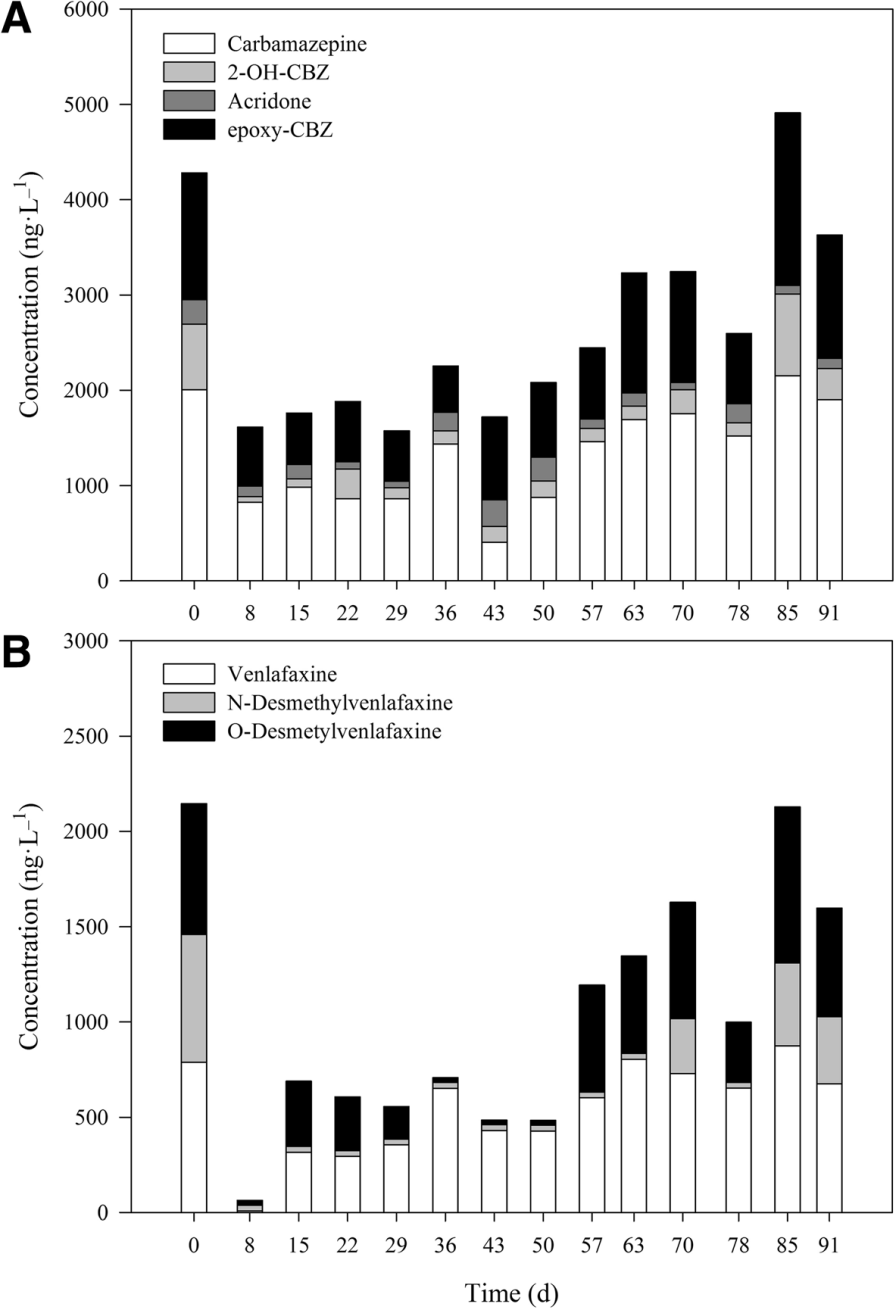
Concentration profile of carbamazepine and its transformation products (**a**) and venlafaxine and its transformation products (**b**) during the treatment

Both carbamazepine and venlafaxine are psychiatric drugs widely used and commonly detected in hospital wastewater, WWTP influent and also WWTP effluent, because both compounds are very recalcitrant to bacterial degradation. In fact, carbamazepine and venlafaxine were, in this order, amongst the most detected psychiatric drugs in Europe [17, 26, 34, 35]. CBZ is metabolized in the human liver by a 95% and produces mainly epoxy-carbamazepine (CBZE), which then is transformed to acridone; CBZ transformation also produces 2-hydroxycarbamazepine (2-OH-CBZ), other TPs and conjugated compounds [36]. These human transformation products can be, and were, detected in the influent wastewater. Additionally, acridone, CBZE and 2-OH-CBZ can also be generated as biodegradation products from fungal and activated sludge activities [7, 37]. Further discussion on CBZ degradation pathway on fungi and humans can be found elsewhere [7]. Carbamazepine (CBZ) and its TPs 2-OH-CBZ, acridone and CBZE concentration profiles are presented in Fig. 4a. The long-term operation removed around 50% of the parent compound CBZ up until Day 50, when CBZ concentration started to increase until reaching influent concentration levels. During the time period where CBZ was biodegraded, 2-OH-CBZ, acridone and CBZE were produced by transformation of CBZ, but the treatment still succeeded in achieving good removal values of CBZ TPs up to Day 63. By the end of the treatment, though, CBZ TPs were not efficiently removed, even when no transformation of CBZ was occurring.

Venlafaxine (VEN) is only excreted through the urine by 1–10% unaltered [37]. This means, it is highly metabolized in humans by cytochrome P450 in liver cells producing primarily O-desmethylvenlafaxine (ODV) –with antidepressant activity– and being N-desmethylvenlafaxine (NDV) a minor metabolic pathway [38]. Concentration profiles of venlafaxine and its TPs in the bioreactor are presented in Fig. 4b. NDV was generally completely removed up to Day 63.

VEN and ODV exhibited low concentrations during the first 6 weeks of steady state, which after Day 63 slowly rose up to concentrations similar than those in the influent. It is worth noting that the presence of VEN in water sample can only be attributed to the input from influent whereas ODV can be generated from the transformation of VEN. Taking into account both groups of CBZ + TPs and VEN + TPs, they all followed a similar pattern, with low concentration at the beginning of the treatment, followed by an increase from Day 63. This behavior was similarly described in another fungal operation (56-day long) where removal of psychiatric drugs decreased over time; it was attributed to an inhibition by *Candida* [17]. In this case, as CBZ and CBZE can be removed by both laccase and the intracellular cytochrome P450 [39, 40], the decrease in laccase activity might have affected CBZ removal. Additionally, a synergistic effect was hypothesized between fungal and bacterial enzymes that led to a high removal of VEN in a batch treatment [12]. An increase in CBZ and its TPs degradation was also observed when non-sterile wastewater was treated, in contrast to sterile WW. Therefore, the microbial community might have played a role in the psychiatric drugs removal diminution (further discussed in section Evolution of bacterial and fungal populations in the bioreactor).

### Evolution of bacterial and fungal populations in the bioreactor

The evolution of microbial populations in the liquid matrix was assessed using DGGE and sequencing of prominent bands, while tracking of *Trametes versicolor* in the pellet and liquid matrices was performed by real-time qPCR. DGGE gels are presented in Additional file 1: Figures S2 and S3. A total of 45 bands were recovered from bacteria and 33 bands from fungi, representing 90 and 98% coverage in the quantitative DGGE band matrix, respectively. Unidentified bands were not considered in further analyses.

**Fig. 5:**
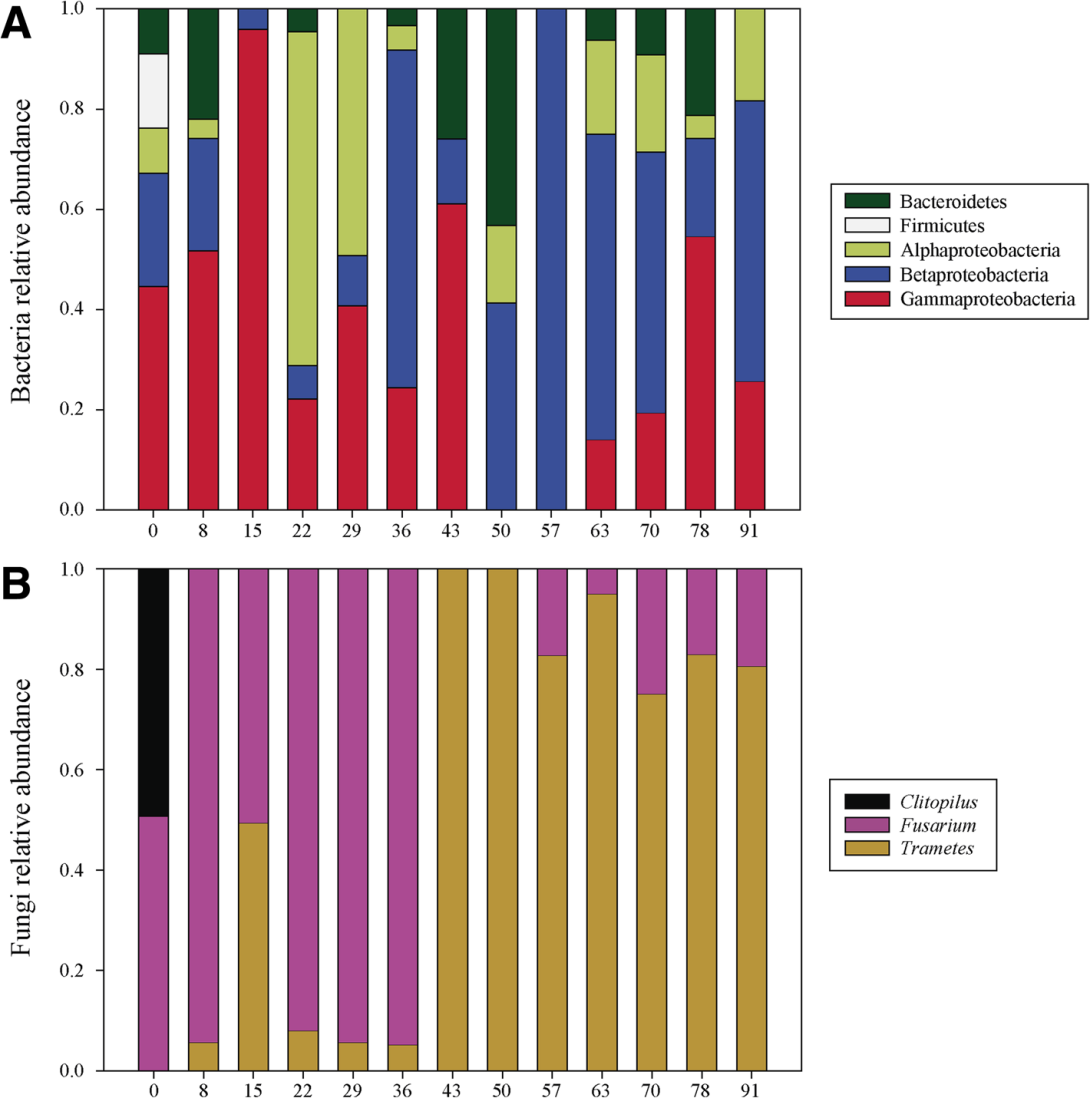
Phylogenetic assignment of bacterial (**a**) and fungal (**b**) sequences from the liquid matrix of the bioreactor. Data is presented in form of relative abundance, previously calculated using the semi-quantitative DGGE matrix and the sequenced bands from DGGE gels. Unidentified bands (10% for bacteria and 2% for fungi) were not represented

Bacterial sequences belonged to 12 different genera (*Acinetobacter, Acutalibacter, Azospirillum, Comamonas, Delftia, Faecalibacterium, Flavobacterium, Luteibacter, Ochrobactrum, Pandoraea, Raoultella* and *Stenotrophomonas*) from the Bacteroidetes, Firmicutes and Proteobacteria phyla. Results displaying the relative abundances of phyla (or classes, in the case of proteobacteria) are presented in Fig. 5. On one hand, Gammaproteobacteria were abundant in the early operation of the bioreactor but oscillated along time displaying three peaks at days 15, 43 and 78. Bacteroidetes behaved similarly, although with lower relative abundances. On the other hand, the Betaproteobacteria was the only group present during all the operation, with particularly high relative abundances (from 0.18 to 1) throughout the mid-late period. This bacterial taxon showed to be well adapted to fungal treatment in a previous work [17]. Firmicutes were only found at the influent and did not colonize the reactor.

Half of the genera (*Acinetobacter*, *Comamonas*, *Delftia*, *Flavobacterium*, *Ochrobactrum* and *Stenotrophomonas*) detected in this study have been found in activated sludge treatments [41, 42]. In fact, both *Comamonas* and *Pandoraea* seemed to be involved in the removal processes, as their abundance during this and previous operations [17] exhibited significative correlations with either psychiatric drugs or antibiotics removal. In the first case, *Comamonas* was negatively correlated to psychiatric drug removal (r = − 0.53 *p* < 0.01). Basically, the genus was abundant when none or low psychiatric drug removal was observed. In the second case, *Pandoraea* had a strong positive correlation with antibiotics removal (r = 0.60 p < 0.01). Bacteria from genus *Pandoraea* are known for their metabolic versatility; some strains are capable of organohalogenate, PAH degradation [43, 44]. While some species are usually found in soil, the genus was described in 2000 [45] from patients suffering cystic fibrosis and it is precisely in the clinical field that many species have been characterized for multiple antibiotic resistance. Antibiotic susceptibility tests revealed resistance of *Pandoraea* isolates to beta-lactams, ciprofloxacin, and even colistin, a last resort antibiotic [45, 46]. While more studies would be necessary to assess to which degree *Pandoraea* is involved in antibiotic degradation, its ability to thrive in presence of antibiotics surely provides fitness advantages in front of other species. By counterpart, the appearance of this genus drew attention to the authors regarding the possibility of pathogenic species being enriched in the liquid matrix and the need to develop further strategies to prevent it.

As a whole, the acidic pH of the reactor, the HRT of 3 days and the presence of *T. versicolor* and its enzymes and microbial products might have affected the bacterial composition developing in the bioreactor. The loss of psychiatric drugs removal from Day 57 onwards could be attributed to the bacterial shift that led to a major abundance of Betaproteobacteria over the other groups. Even if there was no degradation of psychiatric drugs by bacteria in an earlier period, they may have played a role in the further degradation of fungal metabolites, thereby allowing an increased reactor removal capacity [47].

**Fig. 6:**
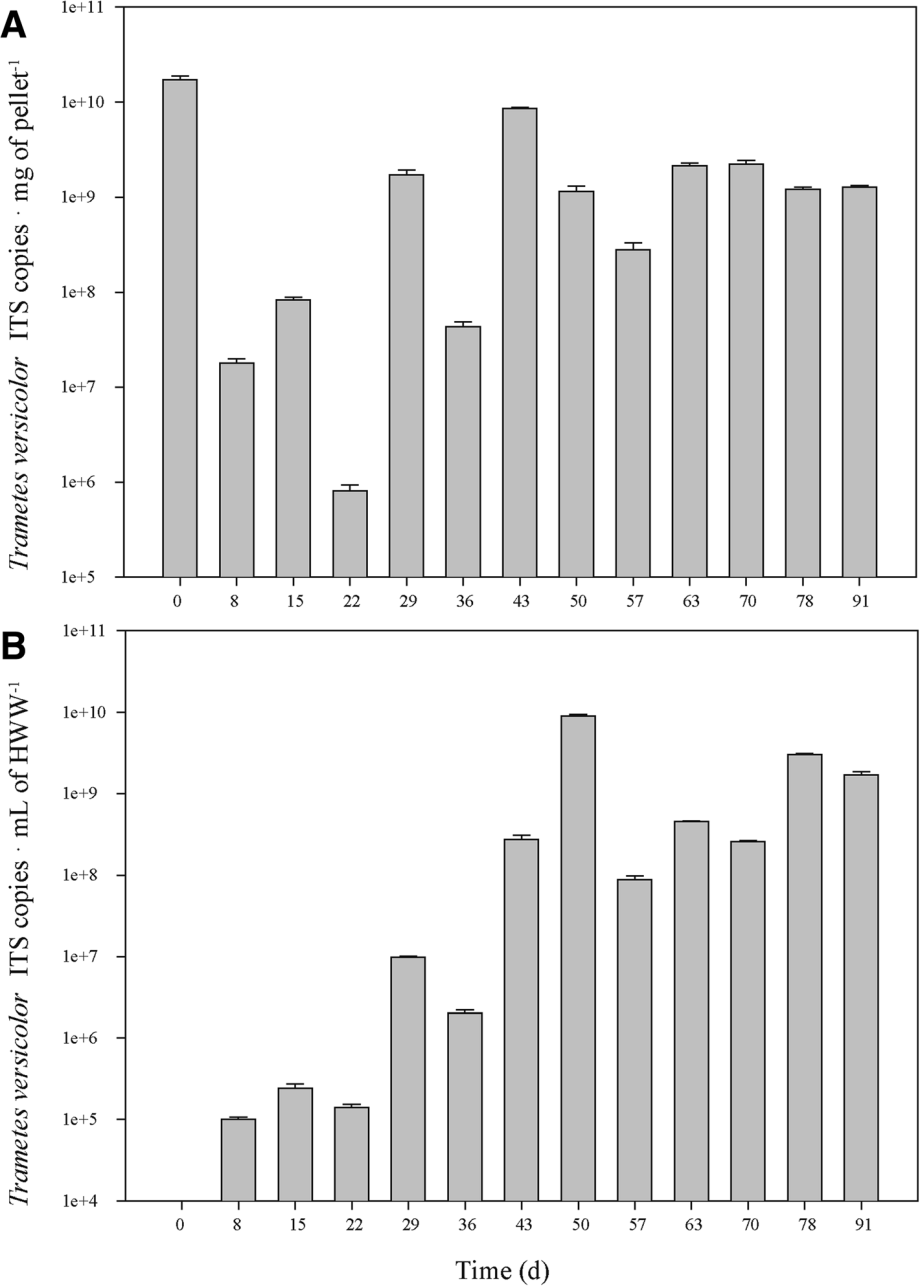
Real-time qPCR quantification of *Trametes versicolor* ITS copies during the bioreactor operation in the pellets (**a**) and liquid matrix (**b**). Standard deviation of qPCR reaction triplicates is included. Liquid matrix sample from day 0 did not amplify

In regards to fungal populations (Fig. 5), although many fungi are typically present in wastewater [48], the semi-quantitative approach DGGE + sequencing identified three fungal genera in the liquid fraction of the bioreactor: *Clitopilus* (Basidiomycota) *Trametes* (Basidiomycota) and *Fusarium* (Ascomycota). Additionally, to further understand the system, the concentration of *Trametes versicolor* in the pellets and liquid matrix was determined through the quantitative, more sensible approach of real-time qPCR. *T. versicolor* ITS copies per mg of pellet and per mL of HWW are presented in Fig. 6a and b, respectively.

*Clitopilus* was only found at the beginning and did not colonize the liquid fraction of the reactor at the conditions of operation. Contrarily, *Fusarium* rapidly colonized the liquid fraction up to a relative abundance of ≥0.92 until Day 43, when the concentration of *Trametes* in the liquid steadily rose from 10^6^ to 10^9^ ITS copies · mL^− 1^ due to the breakdown of pellets into free, dissolved mycelium (Fig. 6). A sporadic increase in turbidity and appearance of pink pigmentation was observed between days 33 and 37, corresponding with the appearance and increase in abundance of the Gammaproteobacteria *Luteibacter*. Recent studies reported the appearance of pink-hued contaminant strains belonging to this genus [49]. Interestingly, this event occurred during the same period when *Trametes* overtook the dominance of the bioreactor. Although the same pigmented bacterium might have antagonistically interacted with *Fusarium*, ITS copies of *Trametes versicolor* in the liquid matrix were increasing since day 0. The biomass renovation of Day 37 helped in outcompeting *Fusarium* for the rest of the operation, observed in the DGGE with relative abundances ranging between 0.75–0.95 and confirmed with the qPCR, with a steady concentration between 9 × 10^7^ and 2 × 10^9^ mL HWW^− 1^. Weekly partial biomass renovations were most likely a major contributor to the predominance of *T. versicolor* throughout the operation. *Fusarium* is a genus of filamentous fungi which includes plant pathogens, but has been studied for dye and steroid degradation [50, 51]. Therefore, its ability and role in the removal of PhACs during the operation could not be disregarded. *T. versicolor* concentration in the pellets (Fig. 6) was maintained since day 29 around 10^9^ ITS copies per mg of pellet. In fact, it seemed that the partial biomass restoration strategy produced a balance between Trametes in the form of free mycelium (10^9^ ITS copies · mL-^1^) and in the shape of pellet (10^9^ ITS copies mg pellet^− 1^) after Day 36.

Finally, to our knowledge this is the first time that the operation has been kept for such a long time. Indeed, a long-term fungal operation to treat non-sterile hospital wastewater (with high loads of PhACs) has been demonstrated for the first time. The fungal approach allowed the removal of the vast majority of compounds from the wastewater during a period of 91 days. The fungal removal capability for most compounds was confirmed despite of the presence of native wastewater microorganisms. The treatment significantly reduced the pharmaceutical load of 4 PhACs listed in the initial watch list of the EU Commission (carbamazepine, clarithromycin, ibuprofen and sulfamethoxazole) [52], and 18 compounds listed in the priority list of the Global Water Research Coalition [32]. Average removal of PhACs was around 80%, with the exception of carbamazepine, bezafibrate, hydrochlorothiazide, metoprolol and trimethoprim (around 50% removal). Increasing the HRT or the fungal concentration could help in improving the removal of pharmaceuticals.

## Conclusions

A long term of fungal treatment removing PhACs from real hospital wastewater has been demonstrated for the first time. The fungus confirmed the ability of bioremediating most compounds from this heavily contaminated matrix. With the suitable strategic conditions is possible to maintain the fungal activity for 90 days. Furthermore, the balance between *Trametes* as a pellet and free mycelium was observed by qPCR. A negative interaction between Gammaproteobacteria *Luteibacter* and WRF *T. versicolor* was described for the first time, although no effect on laccase production or removal efficiency was noted. At the same time *Pandoraea* was identified as a putative antibiotic remover.

## Additional file

Additional file 1: **Table S1**. Physicochemical characterization of the hospital wastewater. **Table S2**. PhACs analysed in the raw HWW and after the pretreatment with coagulation-floculation. **Table S3**. Sequence information from the bacterial DGGE bands obtained. **Table S4**. Sequence information from the fungal DGGE bands obtained. **Figure S1**. pH and temperature profile of the reactor during the treatment. Data was logged every 5 minutes. **Figure S2**. Bacterial DGGE band profiles from the bioreactor operation. Sequenced bands of highest quality are indicated (▲) along with their phylotype (A–S) described in **Table S3**. **Figure S3**. Fungal DGGE band profiles from the bioreactor operation. Sequenced bands of highest quality are indicated (▲) along with their phylotype (A–D) described in **Table S4**. (DOCX 871 kb)
