# Supplementary material for: Long-term continuous treatment of non-sterile real hospital wastewater by Trametes versicolor
Source: J Biol Eng. 2019 May 29;13:47. doi: 10.1186/s13036-019-0179-y (PMC6542094; doi:10.1186/s13036-019-0179-y)
Supplement: Supplementary file 1 — Table S1. Physicochemical characterization of the hospital wastewater. Table S2. PhACs analysed in the raw HWW and after the pretreatment with coagulation-floculation. Table S3. Sequence information from the bacterial DGGE bands obtained. Table S4. Sequence information from the fungal DGGE bands obtained. Figure S1. pH and temperature profile of the reactor during the treatment. Data was logged every 5 minutes. Figure S2. Bacterial DGGE band profiles from the bioreactor operation. Sequenced bands of highest quality are indicated (▲) along with their phylotype (A–S) described in Table S3. Figure S3. Fungal DGGE band profiles from the bioreactor operation. Sequenced bands of highest quality are indicated (▲) along with their phylotype (A–D) described in Table S4. (DOCX 871 kb) [file 13036_2019_179_MOESM1_ESM.docx]

**Supporting information for**

Long-term continuous treatment of non-sterile real hospital wastewater by Trametes versicolor

*Josep Anton Mir-Tutusaus^a^, Eloi Parladé^b^, Adrián Jaén-Gil^c^, Damià Barceló^c^, Sara Rodriguez-Mozaz^c^, Maira Martinez-Alonso^b^, Núria Gaju^b^, Glòria Caminal^d^, Montserrat Sarrà^a^**

^a^Departament d’Enginyeria Química Biològica i Ambiental, Escola d’Enginyeria, Universitat Autònoma de Barcelona, 08193 Bellaterra, Barcelona, Spain

^b^Departament de Genètica i Microbiologia, Universitat Autònoma de Barcelona, 08193 Bellaterra, Barcelona, Spain

^c^Catalan Institute for Water Research (ICRA), Scientific and Technological Park of the University of Girona, H2O Building, Emili Grahit 101, 17003 Girona, Spain

^d^Institut de Química Avançada de Catalunya (IQAC) CSIC. Jordi Girona 18-26, 08034 Barcelona, Spain

The supporting information file contains 4 tables and 3 figures. This file is 9 pages long.

Table S1. Physicochemical characterization of the hospital wastewater

|  | Non flocculated | Flocculated |
| --- | --- | --- |
| pH | 8.3 | 8.9 |
| Conductivity (mS·cm^-1^) | 2.1 | 1.9 |
| Absorbance at 650 nm | 0.239 | 0.082 |
| Chloride (mg Cl·L^-1^) | 343.0 | 359.9 |
| Sulphate (mg S·L^-1^) | 287.7 | 288.4 |
| Nitrate (mg N·L^-1^) | n.d. | n.d. |
| Phosphate (mg P·L^-1^) | 3.33 | 1.20 |
| Ammonia (mg N · L^-1^) | 9.8 | 10.0 |
| TSS (mg·L^-1^) | 276 | 73 |
| COD (mg O_2_·L^-1^) | 507 | 128 |

.

Table S2. PhACs analysed in the raw HWW and after the pretreatment with coagulation-floculation.

Table S2 continuation

Table S3. Sequence information from the bacterial DGGE bands obtained.

|  | | | | |
| --- | --- | --- | --- | --- |
| **Phylotype** | **Band code** | **Closest related sequence (% similarity)** | **Accession number** | **Phylum^a^** |
| A | 3 | *Comamonas* (98‒100) | KX279654 | Betaproteobacteria |
| B | 5 | *Delftia* (100) | MF156902 | Betaproteobacteria |
| C | 10 | *Delftia* (97) | KX980470 | Betaproteobacteria |
| D | 12 | *Faecalibacterium* (96) | AY169429 | Firmicutes |
| E | 13 | *Acinetobacter* (97) | JN849077 | Gammaproteobacteria |
| F | 14 | *Acutalibacter* (97) | CP021422 | Firmicutes |
| G | 16 | *Paraburkholderia* (96) | KY992888 | Betaproteobacteria |
| H | 19 | *Pandoraea* (100) | CP010431 | Betaproteobacteria |
| I | 21 | *Stenotrophomonas* (97) | MF442269 | Gammaproteobacteria |
| J | 22,38,41 | *Raoultella* (98‒100) | MF455198 | Gammaproteobacteria |
| K | 23 | *Raoultella* (99‒100) | MF429591 | Gammaproteobacteria |
| L | 24,26 | *Ochrobactrum* (100) | LC150701 | Alphaproteobacteria |
| M | 27,45 | *Pandoraea* (99) | CP010897 | Betaproteobacteria |
| N | 28,30,33,34 | *Luteibacter* (100) | KY938100 | Gammaproteobacteria |
| O | 31 | *Burkholderia* (100) | MF383417 | Betaproteobacteria |
| P | 32 | *Pedobacter* (96) | EF204468 | Bacteroidetes |
| Q | 36 | *Stenotrophomonas* (100) | KY910087 | Gammaproteobacteria |
| R | 39, 40 | *Azospirillum* (99) | CP012406 | Alphaproteobacteria |
| S | 44 | *Flavobacterium* (98) | FJ447541 | Bacteroidetes |
| ^a^ Sequences belonging to the Proteobacteria phylum are presented at the class level. | | | | |

Table S4. Sequence information from the fungal DGGE bands obtained.

|  | | | | |
| --- | --- | --- | --- | --- |
| **Phylotype** | **Band code** | **Closest related sequence (% similarity)** | **Accession number** | **Phylum** |
| A | 2, 22, 26, 27, 36 | Fusarium (100) | KY582114 | Ascomycota |
| B | 3, 4, 6, 19, 21, 34, 35 | Trametes (100) | KY949632 | Basidiomycota |
| C | 23 | Fusarium (100) | KU361576 | Ascomycota |
| D | 29 | Fusarium (97) | KT269793 | Ascomycota |
|  | | | | |


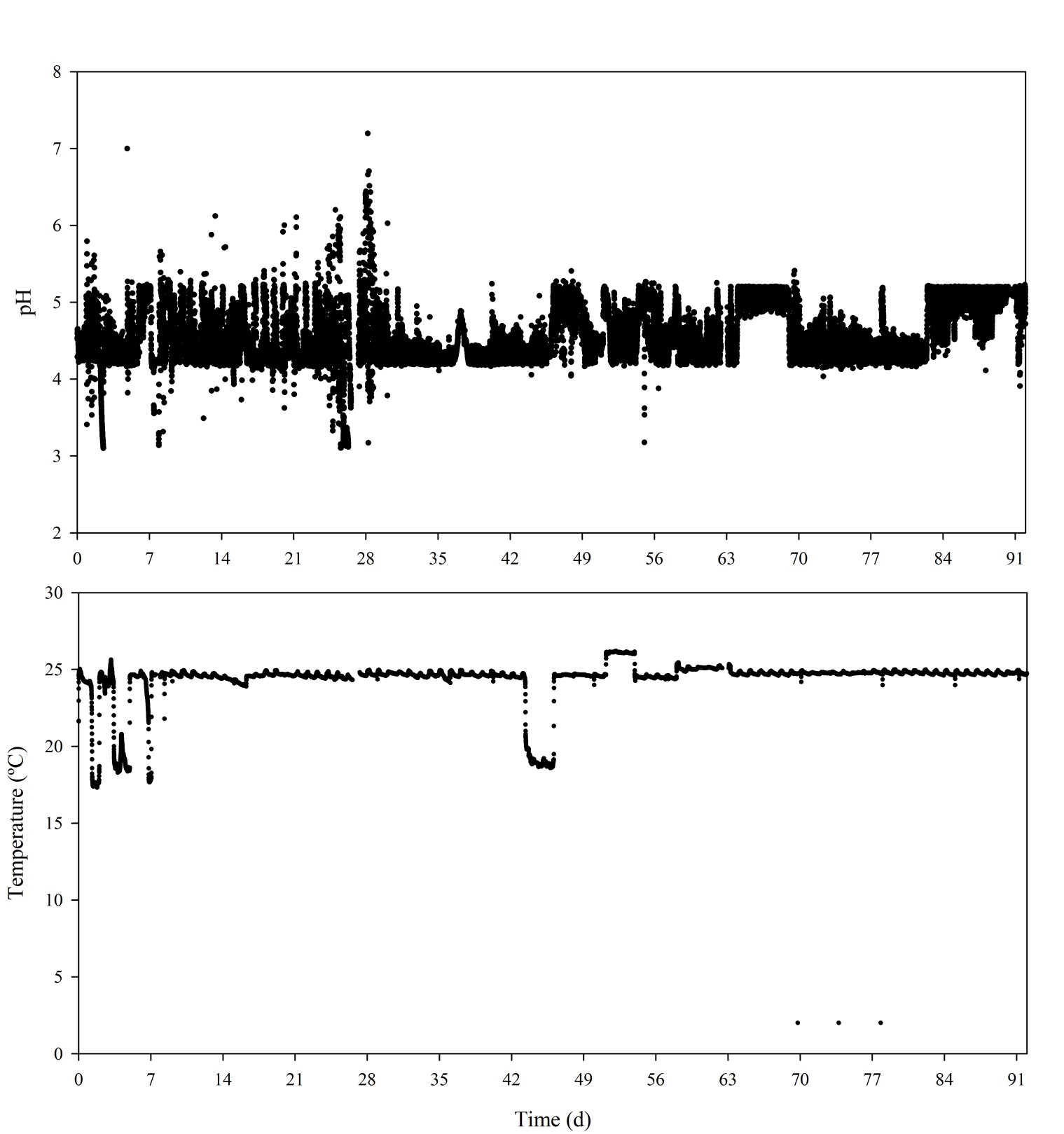


Figure S1. pH and temperature profile of the reactor during the treatment. Data was logged every 5 minutes.


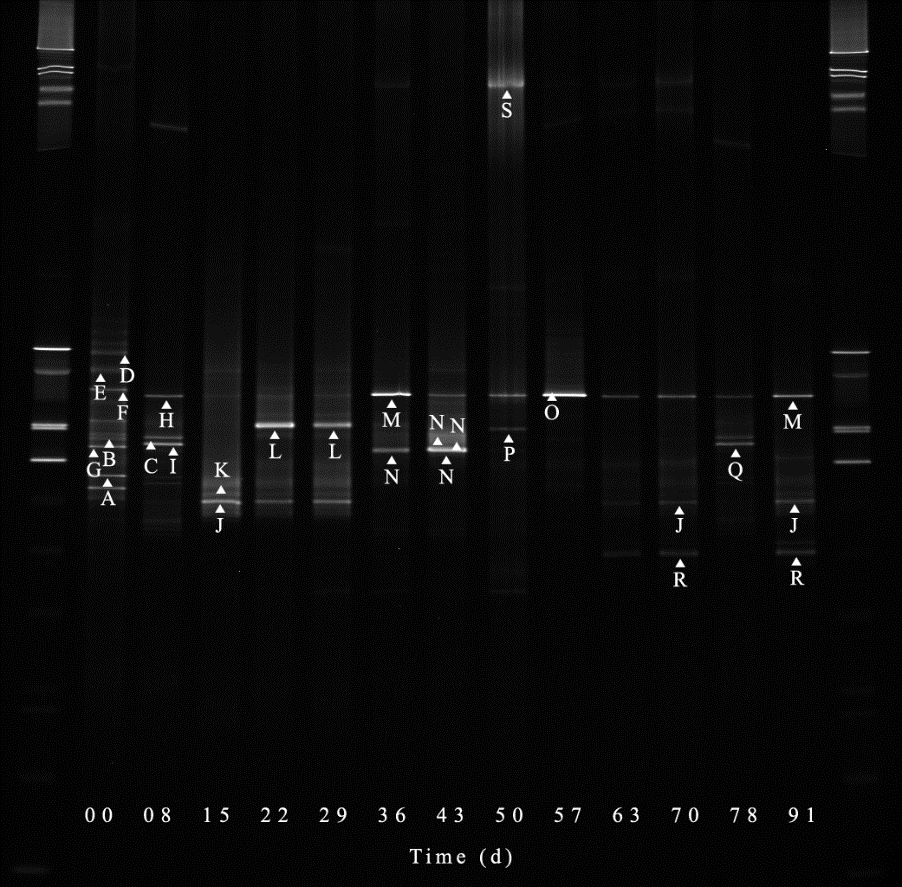


Figure S2. Bacterial DGGE band profiles from the bioreactor operation. Sequenced bands of highest quality are indicated (▲) along with their phylotype (A‒S) described in Table S3.


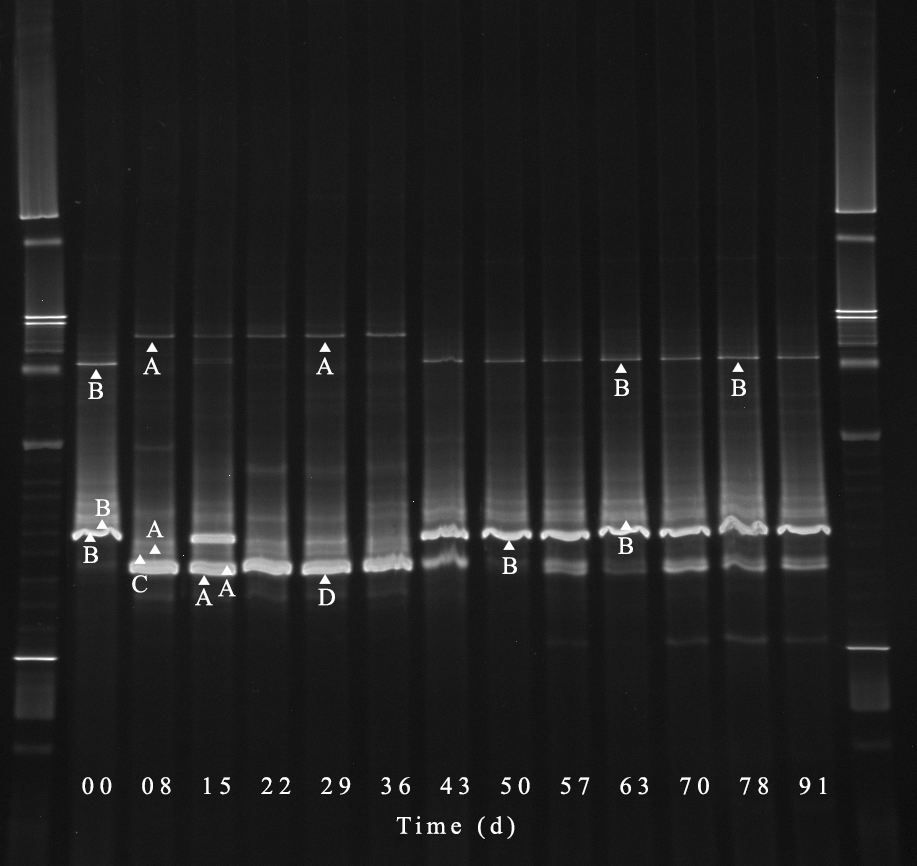


Figure S3. Fungal DGGE band profiles from the bioreactor operation. Sequenced bands of highest quality are indicated (▲) along with their phylotype (A‒D) described in Table S4.
